# Greater accordance with the Dietary Approaches to Stop Hypertension dietary pattern is associated with lower diet-related greenhouse gas production but higher dietary costs in the United Kingdom^1^,^2^

**DOI:** 10.3945/ajcn.114.090639

**Published:** 2015-04-29

**Authors:** Pablo Monsivais, Peter Scarborough, Tina Lloyd, Anja Mizdrak, Robert Luben, Angela A Mulligan, Nicholas J Wareham, James Woodcock

**Affiliations:** 3 UKCRC Centre for Diet and Activity Research, MRC Epidemiology Unit, University of Cambridge, Cambridge, United Kingdom;; 4 British Heart Foundation Health Promotion Research Group, Nuffield Department of Population Health, University of Oxford, Oxford, United Kingdom; and; 5 Strangeways Research Laboratories, Department of Public Health and Primary Care, University of Cambridge, Cambridge, United Kingdom

**Keywords:** climate, diet quality, food prices, prevention, public health

## Abstract

**Background:** The Dietary Approaches to Stop Hypertension (DASH) diet is a proven way to prevent and control hypertension and other chronic disease. Because the DASH diet emphasizes plant-based foods, including vegetables and grains, adhering to this diet might also bring about environmental benefits, including lower associated production of greenhouse gases (GHGs).

**Objective:** The objective was to examine the interrelation between dietary accordance with the DASH diet and associated GHGs. A secondary aim was to examine the retail cost of diets by level of DASH accordance.

**Design:** In this cross-sectional study of adults aged 39–79 y from the European Prospective Investigation into Cancer and Nutrition–Norfolk, United Kingdom cohort (*n* = 24,293), dietary intakes estimated from food-frequency questionnaires were analyzed for their accordance with the 8 DASH food and nutrient-based targets. Associations between DASH accordance, GHGs, and dietary costs were evaluated in regression analyses. Dietary GHGs were estimated with United Kingdom-specific data on carbon dioxide equivalents associated with commodities and foods. Dietary costs were estimated by using national food prices from a United Kingdom–based supermarket comparison website.

**Results:** Greater accordance with the DASH dietary targets was associated with lower GHGs. Diets in the highest quintile of accordance had a GHG impact of 5.60 compared with 6.71 kg carbon dioxide equivalents/d for least-accordant diets (*P* < 0.0001). Among the DASH food groups, GHGs were most strongly and positively associated with meat consumption and negatively with whole-grain consumption. In addition, higher accordance with the DASH diet was associated with higher dietary costs, with the mean cost of diets in the top quintile of DASH scores 18% higher than that of diets in the lowest quintile (*P* < 0.0001).

**Conclusions:** Promoting wider uptake of the DASH diet in the United Kingdom may improve population health and reduce diet-related GHGs. However, to make the DASH diet more accessible, food affordability, particularly for lower income groups, will have to be addressed.

## INTRODUCTION

Diets that are consistent with the Dietary Approaches to Stop Hypertension (DASH)^6^ are associated with reduced cardiometabolic risk and better health outcomes. The design of the DASH diet, which is rich in plant foods and low-fat dairy products and relatively low in fats and sugars, was predicated on the notion that the health-promoting effects of diet arise from the *overall diet* rather than from individual foods or nutrients (1). In randomized trials, DASH dietary patterns were shown to lower blood pressure in persons with hypertension and prehypertension, even without sodium reduction (2, 3). The beneficial effects of the DASH diet were observed within 2 wk and, when combined with sodium reduction, were similar in magnitude to the results of pharmaceutical trials (1). Observational studies found associations between DASH-accordant diets and reduced weight gain (4), lower incidence of stroke (5), heart failure (6), and fatal cardiovascular disease more generally (7). Accordance to DASH was also associated with reduced risk of type 2 diabetes (8) and colorectal cancer (9).

The characteristics of the DASH diet that promote health may also have implications for its environmental impact. The DASH and other plant-centered diets may be an important and effective way to reduce the production of greenhouse gases (GHGs) associated with food consumption. Most of the climate impact of food production is due to methane (emitted by ruminant animals during digestion) and nitrous oxide (released from the land during tilling) (10). Over a 100-y period, methane is 25 times more potent a GHG than carbon dioxide, and nitrous oxide is 300 times more potent (11). Research on the relation between diet and GHGs is in its infancy, but modeling studies have suggested that reducing consumption of meat and other animal-derived foods can simultaneously reduce the GHG impact of the diet and reduce risk of chronic disease (12, 13).

The potential population health and climate-related cobenefits of the DASH diet provide sound justification to promote the diet’s wider adoption, but several barriers may exist to its uptake. In particular, the adoption of DASH diets may be affected by food prices. A recent study found that for most adults in the United States, consuming a DASH-accordant diet was associated with higher dietary costs (14), which is broadly consistent with earlier work reporting that healthier diets tend to carry a price premium (15). Identifying affordable healthy diets is an important step toward increasing the wider adoption of dietary recommendations.

This study combined dietary data with food-level GHG and price data to examine the GHG impact and cost of diets in relation to their accordance to the DASH diet pattern in a large, population-based sample of adults in the United Kingdom.

## METHODS

### Subjects

We used data from the population-based European Prospective Investigation into Cancer and Nutrition cohort study in Norfolk, United Kingdom (EPIC-Norfolk). Recruitment was based on registers of general practices in the county. Participants were aged 39–79 y at the time of entry (1993–97), when they were weighed and measured and completed questionnaires and dietary assessments. Of the 25,639 adults recruited at baseline, our analyses were restricted to the 24,293 participants who had valid dietary data (described below). Volunteers received no dietary counseling or advice in this observational study. All volunteers gave written informed consent, and the study was approved by the Norwich district ethics committee.

### Dietary assessment

Participants reported their usual food intake during the previous year by completing a 130-item, semiquantitative food-frequency questionnaire (FFQ) described previously (16). Intake for each item was reported in frequencies ranging from “never or less than once/month” to “6 times per day or more.” The servings were specified in terms of units or common portions (e.g., one apple, one slice of bread) or household measures (e.g., glass, cup, or spoon). Further questions allowed us to categorize breakfast cereals consumed for the purpose of estimating overall consumption of whole grains. Similarly, questions on milk allowed us to estimate intakes in the low-fat dairy category of the DASH score. The FFQ data were processed by using the FFQ EPIC Tool for Analysis (17), software based on the earlier analysis system (16), to estimate average daily nutrient and energy intakes. Dietary data were deemed implausible when energy estimates fell below or exceeded a range of acceptable estimated energy requirements based on age, weight, and sex as described previously (16). For further details on the nutritional analysis of the EPIC FFQ, see Welch et al. (16).

### Accordance to the DASH dietary pattern

Accordance to the DASH dietary pattern was based on a score similar to one previously applied to the Nurses’ Health Study FFQ (5). That score was based on consumption of 8 food groups and nutrients, adjusted for energy. The original food groups and nutrients used were fruits, vegetables, nuts and legumes, whole grains, low-fat dairy, red and processed meats, sweetened beverages, and sodium. We modified the sweetened beverages group to better account for the multiple food sources of added sugar in the diets of the EPIC-Norfolk sample. The revised category included a broader range of foods high in added sugars. The 8 DASH food groups are presented and described in **Table 1**. More details on assessing accordance to DASH, including changes to the scoring, are described in the **Supplemental Materials**. The DASH accordance score has a minimum value of 8 and a maximum value of 40.

**TABLE 1:** Food groups used to assess dietary accordance to the Dietary Approaches to Stop Hypertension

Food group	Foods included	Scoring^1^
Fruits	All fresh, dried, and tinned fruit and fruit juice	+
Vegetables	All vegetables, including fresh, canned, and frozen peas and green beans, but not potatoes	+
Nuts and legumes	Dried beans and dried peas, nuts, peanut butter, baked beans, and soya food	+
Whole grains	Whole-grain bread, crisp bread, brown rice, whole-meal pasta, porridge, cereals	+
Low-fat dairy products	Yogurt, low-fat cottage cheese, low-fat margarine, low-fat milk	+
Red and processed meats	Beef, beef burgers, pork, lamb, bacon, ham, corned beef, sausages	−
Foods high in added sugars	Confectionary, sweetened grain-based products (sweet biscuits, sweet pastries, and buns); sugar-sweetened carbonated drinks; sugar added to hot drinks and breakfast cereals	−
Sodium	Dietary sodium	−

1 Scoring of “+” indicates food groups that are positively scored; “−” indicates food groups that are negatively scored (i.e., greater consumption of these groups is associated with a lower score).

### Calculation of dietary GHG emissions

For each of the 289 food codes represented in the FFQ’s food and nutrient database, we estimated the GHG emissions, measured as kg of CO_2_ equivalents (CO_2_eq), as the quantity of 3 GHGs weighted by their global warming potential over a 100-y period. The GHGs were carbon dioxide (weighted as 1), methane (weighted as 25), and nitrous oxide (weighted as 298), per 100 g of food. This method was adapted from a previous investigation of the health impact of applying a carbon tax to foods in the United Kingdom (18). The source document for GHG parameters was Audsley et al. (19), which estimated comparable GHG emissions for 94 food commodities consumed in the United Kingdom. These estimates incorporated the life cycle of food commodities from the earliest stages of production to the retail distribution center. The GHG emissions for the 289 food codes were then constructed from these 94 parameters by using representative ingredient lists and adjustments for density, and the diet-level variable derived was kg CO_2_eq/d. More details on the mapping of GHG values to the foods in the FFQ instrument are provided in the **Supplemental Materials**.

### Cost of diets

The monetary cost of the reported diets was estimated by attaching a food price vector to the FFQ’s nutrient composition database as described previously (20). Retail prices for each of the 289 component food items in the FFQ were obtained by using standardized and published price collection methods (21). In brief, each food and drink in the FFQ was priced by using MySupermarket.com, a website for comparing supermarket food prices nationwide in the United Kingdom. For each of the 289 items in the FFQ, the lowest, nonsale price was selected from among the 5 nationwide retailers represented on the website at that time (June 2012): Tesco, Sainsbury’s, Asda, Waitrose, and Ocado, which together had a 68% market share in 2012 (22).

For packaged goods, including most fresh produce, the package size in the middle of the range was typically selected. If only 2 package size options were available, the larger was chosen. As described previously, prices were adjusted for preparation and waste (21) to yield an adjusted food price of £/100-g edible portion. The addition of this new variable to the EPIC-Norfolk food and nutrient database (16) allowed the derivation of dietary cost for each participant. The variable associated with each individual’s diet was cost per day (£/d).

### Analytic approach

We used general linear models to evaluate the DASH accordance score by demographic and socioeconomic strata, which allowed adjustment for age, sex, and dietary energy. DASH accordance by sex was evaluated in models adjusting for age. General linear models were also used to produce covariate-adjusted estimates of GHG emissions and costs of diets by quintile of DASH accordance. In all models, ANOVA was used to detect statistically significant overall heterogeneity across groups. In analysis of quintiles of DASH accordance, trend tests were conducted to test for systematic increase/decrease across groups, and pairwise tests were conducted to examine the difference between extreme groups. All statistical analyses were conducted with SPSS version 19 (SPSS Inc.).

## RESULTS

### Sample characteristics

Our sample had a mean age of 59 y and was 55% female, with 80% reporting good/excellent general health and 46% never having smoked. For the whole sample, 13% were educated to degree level, and the highest 2 occupational social classes made up 44% of the sample, with a greater proportion of men than women in the higher socioeconomic categories. Women reported consuming fewer total calories than men (1928 vs. 2191 kcal/d, respectively). Detailed demographic characteristics of the sample are provided in **Supplemental Table 1**.

### Dietary accordance with the DASH

DASH accordance scores were normally distributed over the full range (8–40) with an overall mean score of 24. In **Table 2**, the mean overall DASH diet score by demographic and socioeconomic characteristics is provided. Women showed higher DASH accordance than did men, and in age and sex-adjusted models, DASH accordance was higher for people of higher socioeconomic status, whether indicated by occupational social class or by educational attainment. Current smokers had significantly less DASH-accordant diets than both never and former smokers.

**TABLE 2:** DASH diet scores by demographic and socioeconomic characteristics of British adults in the European Prospective Investigation into Cancer and Nutrition–Norfolk cohort^1^

	*n*	DASH diet score (scale 8–40)
Total	24,293	23.9 (23.9, 24.0)^2^
Sex^3^		
Men	10,980	23.1 (23.0, 23.2)
Women	13,313	24.7 (24.7, 24.8)
*P* value^4^		<0.0001
Occupational social class^5^		
Unskilled	1667	22.5 (22.2, 22.8)
Partly skilled	8747	23.1 (22.9, 23.3)
Skilled occupations—manual	3952	23.3 (23.2, 23.4)
Skilled occupations—nonmanual	5478	24.0 (23.9, 24.1)
Managerial and technical	3143	24.5 (24.4, 24.6)
Professional	806	25.1 (24.9, 25.3)
*P* value^4^		<0.001
Educational attainment,^6^ education y		
<11	8871	23.0 (22.9, 23.1)
11–13	2487	23.8 (23.7, 24.0)
>13 to <16	9798	24.3 (24.2, 24.3)
≥16	3122	25.6 (25.4, 25.7)
*P* value^4^		<0.0001
Smoking status^7^		
Current smoker	2775	21.9 (21.7, 22.1)
Former smoker	10,172	24.2 (24.1, 24.3)
Never smoker	11,147	24.2 (24.1, 24.3)
*P* value^4^		<0.0001

1 DASH, Dietary Approaches to Stop Hypertension.

2 Mean; 95% CI in parentheses (all such values).

3 Age adjusted.

4 *P* value for heterogeneity based on ANOVA.

5 Based on Registrar General’s classification, age and sex adjusted, 500 missing cases.

6 Age and sex adjusted, 15 missing cases.

7 Total of 199 missing cases.

### Dietary GHG emissions

**FIGURE 1:**
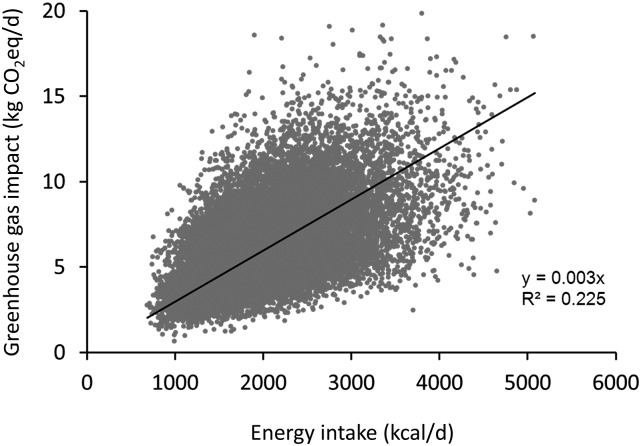
Scatterplot illustrating the relation between estimated energy intake and estimated GHG impact, in kg of CO_2_eq/d. Sample *n* = 24,293 men and women. For clarity, the vertical axis is capped at 20 kg CO_2_eq/d, excluding from the figure 25 cases with GHG estimates ranging from 20.3 to 48.5 kg/d. Least squares regression line forced through the origin, *P* < 0.0001. CO_2_eq, carbon dioxide equivalents; GHG, greenhouse gas.

Dietary GHG emissions in this sample, estimated in kg of CO_2_eq, followed a log-normal distribution. The mean ± SD estimated crude GHG impact of diets was 6.3 ± 2.5 kg CO_2_eq/d, with the diets of women having a lower impact than those of men (5.9 ± 2.2 vs. 6.7 ± 2.6 kg/d, respectively). Differences between women and men were partly driven by differences in energy intake. The scatterplot in **Figure 1** illustrates that, although GHG impact was positively associated with total reported energy intake, GHG impact varied substantially at any level of intake. For instance, at 2000 kcal, emissions ranged from ∼2 kg to >12 kg CO_2_eq/d. The slope of a least squares linear regression line indicated a mean increase of 3 kg CO_2_eq/1000 kcal of dietary energy.

### Costs of diets

As with GHG impact, diet costs followed a log-normal distribution, ranging from £0.84 to £18.10/d and an overall mean ± SD of £4.06 ± 1.22/d. Men’s diets were slightly more costly (£4.12 ± 1.24/d) than women’s (£4.01 ± 1.20/d), partly reflecting higher energy intakes among men.

### Overall DASH accordance, GHG impact, and costs of diets

DASH accordance scores were systematically associated with both GHG impact and dietary costs. GHG emissions and dietary cost for each quintile of DASH accordance are shown in **Table 3**. There was a negative, monotonic association between DASH accordance and GHG impact. Adults in the highest quintile [quintile 5 (Q5), closest accordance with DASH] consumed diets with a mean GHG impact that was 1.1 kg CO_2_eq/d (16%) lower than diets in the lowest quintile [quintile 1 (Q1)]. Diet cost showed a positive, monotonic association with DASH accordance, and diets in Q5 of DASH accordance were £0.67/d (18%) more costly than diets in Q1. The association between DASH accordance and diet cost was modified by sex (*P*-interaction = 0.018), so we also produced sex-stratified estimates. The analyses, shown in **Supplemental Table 2**, indicated that the DASH accordance–dietary cost relation was stronger in women than in men. The association between DASH accordance and GHG did not appear to be modified by sex (*P*-interaction = 0.170).

**TABLE 3:** Energy intake, greenhouse gas (CO_2_ equivalents), and diet cost for quintiles of overall DASH accordance^1^

Quintile of DASH accordance (DASH score range)	*n*	Energy intake, kcal/d	CO_2_ equivalents, kg/d	Dietary cost, £/d
Q1 (8–20, lowest accordance)	5914	2094 (2079, 2109)^2^	6.71 (6.66, 6.76)	3.74 (3.72, 3.76)
Q2 (21–23)	5359	2026 (2011, 2042)	6.48 (6.42, 6.53)	3.95 (3.92, 3.97)
Q3 (24–25)	3838	2004 (1985, 2022)	6.27 (6.20, 6.34)	4.08 (4.05, 4.10)
Q4 (26–28)	4914	2038 (2022, 2055)	6.09 (6.03, 6.15)	4.20 (4.18, 4.23)
Q5 (29–39, highest accordance)	4268	2126 (2109, 2144)	5.60 (5.54, 5.67)	4.41 (4.38, 4.43)
Difference Q5/Q1, %		+1.5	−16.5	+17.9
*P* value, Q1 vs. Q5^3^		0.007	<0.0001	<0.0001
*P*-trend^4^		0.08	<0.0001	<0.0001

1 All estimates are adjusted for age and sex. Greenhouse gas and cost estimates are also adjusted for dietary energy. Data from the European Prospective Investigation into Cancer and Nutrition–Norfolk cohort. DASH, Dietary Approaches to Stop Hypertension; Q, quintile.

2 Mean; 95% CI in parentheses (all such values).

3 Based on post hoc, pairwise test comparing highest and lowest levels.

4 Based on linear regression with quintiles of DASH accordance treated as a group linear variable.

### Association between accordance to DASH food groups and dietary GHG emissions

The association between DASH accordance and dietary GHG emissions was further explored in regression models that examined accordance to the 8 DASH food groups individually, adjusted for age, sex, and total dietary energy. Accordance with the 8 DASH food groups was differentially associated with emissions, with some groups showing lower, higher, or no association with GHGs (**Table 4**). Accordance with 4 of the 8 food groups (fruit, whole grains, red and processed meat, and dietary sodium) showed a negative association with emissions, implying that achievement of these criteria is associated with lower emissions. The nuts and legumes group had only a weak association with GHG impact. Accordance with the vegetable, low-fat dairy, and foods high in sugars categories was positively associated with GHG impact. Among the 8 food groups, the strongest association was seen for red and processed meat, in which the most accordant diets (Q5) had an associated GHG impact that was 4.3 kg CO_2_eq/d (49.5%) lower than the GHG impact associated with Q1 diets. This was partially offset by vegetables and foods high in sugars, which both showed a GHG impact for Q5 that was about 1 kg CO_2_eq/d (16%) greater than diets in Q1.

**TABLE 4:** Greenhouse gas by quintiles of accordance to food groups in the DASH diet^1^

	Food group, kg CO_2_eq/d							
Quintile of accordance to DASH food and nutrient groups	Fruit	Vegetables	Nuts and legumes	Whole grains	Low-fat dairy foods	Red and processed meat	Foods high in sugars	Dietary sodium
Q1 (lowest accordance)	6.36 (6.30, 6.43)^2^	5.77 (5.71, 5.83)	6.23 (6.17, 6.29)	6.64 (6.58, 6.69)	6.28 (6.22, 6.34)	8.59 (8.55, 8.63)	5.84 (5.78, 5.90)	6.52 (6.46, 6.58)
Q2	6.32 (6.26, 6.38)	6.07 (6.01, 6.12)	6.30 (6.24, 6.36)	6.47 (6.41, 6.53)	6.18 (6.12, 6.24)	7.08 (7.04, 7.13)	6.06 (6.00, 6.12)	6.26 (6.20, 6.32)
Q3	6.25 (6.19, 6.31)	6.33 (6.27, 6.39)	6.40 (6.34, 6.46)	6.37 (6.31, 6.43)	6.23 (6.17, 6.29)	6.04 (5.99, 6.08)	6.25 (6.19, 6.31)	6.26 (6.20, 6.31)
Q4	6.20 (6.14, 6.26)	6.52 (6.46, 6.58)	6.24 (6.18, 6.3)	6.14 (6.08, 6.19)	6.23 (6.17, 6.29)	5.30 (5.26, 5.34)	6.43 (6.37, 6.49)	6.18 (6.12, 6.24)
Q5 (highest accordance)	6.18 (6.12, 6.25)	6.70 (6.64, 6.76)	6.21 (6.15, 6.27)	5.71 (5.66, 5.77)	6.45 (6.39, 6.51)	4.34 (4.29, 4.38)	6.80 (6.74, 6.86)	6.18 (6.12, 6.24)
Difference Q5/Q1, %	−2.8	+16.1	−0.3	−14.0	+2.7	−49.5	+16.4	−5.2
*P* value, Q1 vs. Q5^3^	0.0001	<0.0001	0.595	<0.0001	<0.0001	<0.0001	<0.0001	<0.0001
*P*-trend^4^	<0.0001	<0.0001	0.263	<0.0001	<0.0001	<0.0001	<0.0001	<0.0001

1 Analyses are adjusted for age, sex, and dietary energy. Data from the European Prospective Investigation into Cancer and Nutrition–Norfolk cohort. CO_2_eq, CO_2_ equivalents; DASH, Dietary Approaches to Stop Hypertension; Q, quintile.

2 Mean; 95% CI in parentheses (all such values).

3 Based on post hoc, pairwise test comparing highest and lowest levels.

4 Based on linear regression with quintiles of each food group treated as a group linear variable.

### Association between accordance to DASH food groups and diet cost

As with GHG impact, the accordance to DASH food groups showed different associations with diet cost, with some groups more strongly related to diet cost than others (**Table 5**). Accordance to one DASH food group—red and processed meat—was associated with cost savings, with highest accordance showing dietary costs that were £0.82/d (18%) lower than diet costs in Q1. Greater accordance with 3 food groups was associated with markedly higher diet costs. Highest accordance (Q5) with fruit, vegetables, and foods high in sugars was associated with diets that were 27%, 40%, and 19% more costly than diets in Q1, respectively. The remaining 4 food groups showed either no association with cost (whole grains and dietary sodium) or only a small (1–4%) difference in cost between most- and least-accordant diets (nuts and legumes and low-fat dairy).

**TABLE 5:** Dietary cost by quintiles of accordance to food groups in the DASH diet^1^

	Dietary cost, £/d							
Quintile of accordance to DASH food and nutrient groups	Fruit	Vegetables	Nuts and legumes	Whole grains	Low-fat dairy foods	Red and processed meat	Foods high in sugars	Dietary sodium
Q1 (lowest accordance)	3.63 (3.60, 3.65)^2^	3.41 (3.39, 3.44)	4.00 (3.97, 4.03)	3.97 (3.95, 4.00)	3.88 (3.86, 3.91)	4.53 (4.51, 4.55)	3.75 (3.73, 3.77)	4.13 (4.10, 4.15)
Q2	3.84 (3.81, 3.86)	3.77 (3.75, 3.79)	4.02 (3.99, 4.05)	4.02 (4.00, 4.05)	4.18 (4.15, 4.20)	4.16 (4.14, 4.19)	3.83 (3.81, 3.86)	4.01 (3.99, 4.04)
Q3	4.01 (3.99, 4.03)	4.02 (4.00, 4.05)	4.07 (4.05, 4.10)	4.13 (4.10, 4.15)	4.05 (4.03, 4.08)	3.96 (3.94, 3.99)	3.99 (3.97, 4.02)	4.00 (3.97, 4.02)
Q4	4.17 (4.15, 4.20)	4.29 (4.26, 4.31)	4.09 (4.06, 4.11)	4.13 (4.10, 4.16)	4.08 (4.06, 4.11)	3.85 (3.83, 3.88)	4.18 (4.15, 4.20)	4.00 (3.97, 4.02)
Q5 (highest accordance)	4.62 (4.59, 4.64)	4.78 (4.75, 4.80)	4.05 (4.02, 4.07)	3.98 (3.95, 4.00)	4.04 (4.01, 4.06)	3.71 (3.69, 3.74)	4.48 (4.45, 4.50)	4.10 (4.07, 4.12)
Difference Q5/Q1, %	+27.3	+40.2	+1.3	+0.3	+4.1	−18.1	+19.5	−0.7
*P* value, Q1 vs. Q5^3^	<0.0001	<0.0001	0.009	0.936	<0.0001	<0.0001	<0.0001	0.138
*P*-trend^4^	<0.0001	<0.0001	0.0001	0.014	<0.0001	<0.0001	<0.0001	0.106

1 Analyses are adjusted for age, sex, and dietary energy. Data from the European Prospective Investigation into Cancer and Nutrition–Norfolk cohort. DASH, Dietary Approaches to Stop Hypertension; Q, quintile.

2 Mean; 95% CI in parentheses (all such values).

3 Based on post hoc, pairwise test comparing highest and lowest levels.

4 Based on linear regression with quintiles of each food group treated as a group linear variable.

## DISCUSSION

In this report, we provide evidence from a large, population-based study of British adults that dietary patterns that were more accordant with the DASH diet pattern had a lower climate impact, in terms of the GHGs associated with the foods consumed. These findings provide further rationale for promoting the DASH diet more widely in Britain, where prevailing unhealthy diets contribute to excess rates of chronic disease and mortality (23) and where the food system contributes more than one-fifth of all GHGs produced in the United Kingdom (10, 24). Previous reports have pointed to the potential health and climate cobenefits of shifting the population to healthier diets (13, 25–27), but to our knowledge, this is the first study demonstrating the climate impact of diets in relation to accordance to DASH dietary targets.

Not all of the 8 DASH food groups showed a climate benefit. For instance, consumption of diets high in sugar had lower GHG emissions than diets low in sugar (see Table 4). Similar findings have been reported previously (25, 28) and may be because the production of sugar has very low carbon footprint (29), especially when measured per kcal (19). This tension between health and sustainability goals has previously been observed in a study that modeled the effect of a GHG tax and subsidy regimen for foods, which resulted in subsidies for sugar-sweetened beverages and consequent adverse health outcomes (18). Our finding that higher vegetable intake was associated with higher dietary GHGs is consistent with a previous French study showing that some modeled diets with increased vegetable consumption could have higher GHG emissions (30).

Other diet patterns might be associated with even lower dietary GHGs. For example, a recent United Kingdom-based study, using similar methods to those presented here, compared meat eaters, vegetarians, and vegans (31). Relative to moderate meat eaters, those following vegetarian or vegan diets had dietary GHG emissions that were 1.8 kg CO_2_eq/d (32%) or 2.7 kg CO_2_eq/d (49%) lower. A modeling study from the United Kingdom showed that even more dramatic reductions in GHGs were possible if the dietary repertoire was limited to a small number of foods optimized for their nutritional and carbon characteristics (25). By contrast, we found that the most DASH-accordant diets were only 1.1 kg CO_2_eq/d (17%) lower than for the least-accordant diets. The difference in findings indicates that limiting or excluding animal-based foods can result in much lower carbon diets than following the DASH diet, which encourages low-fat dairy and lean meat in moderation (32).

Nevertheless, the evidence base indicating the health benefits of the DASH diet pattern is probably the most robust among dietary patterns, with evidence from clinical trials and observational studies (33). Moreover, the DASH diet was designed to be largely congruent with US norms of food consumption (32), which might make it more acceptable for the British population than other evidence-based diet patterns such as the Mediterranean and Okinawan diets (33, 34).

The DASH diet’s cultural acceptability and its potential to benefit both health and environment are likely to be insufficient to drive its broad adoption. Among the structural barriers to adopting healthier eating habits, the cost of food is significant, particularly in a United Kingdom context, in which food prices rose >30% from 2007 to 2013 (35). This trend has coincided with deterioration of diet quality, particularly among the lowest income groups (36). In this study, we found that the most DASH-accordant diets were 18% more costly than unhealthy diets, with the differential greater for women than for men. This finding is consistent with earlier work indicating a price premium for healthier diets generally (15) and is similar to findings reported in a recent study of dietary intakes in the US population, where the most DASH-accordant diets were 19% more costly than the least-accordant diets (14).

Our analysis of the dietary costs of accordance to DASH food groups is consistent with previous studies, which have found that diets higher in fruit and vegetables and lower in sugar were more costly (37–40). We also found that improving accordance to the nuts and legumes food group was associated with only a modest increase in dietary costs, which is consistent with an earlier US study that suggested that increasing intake of this food group could be an economical way to improve overall diet quality (39). The consistent finding that healthier diets tend to carry a price premium highlights the need to identify components of healthy diets that can be obtained at low cost and to look for ways in which the affordability of healthy diets can be increased, particularly for those on lower incomes.

Our results indicated that accordance with some DASH targets could be improved with no or minimal additional cost to consumers. For instance, increasing intake of whole grains and lowering intake of sodium appeared to be cost-neutral, and lowering intake of red and processed meat could produce substantial cost savings. Aligning these findings with the food group–level analysis of GHG impact can reveal where dietary improvements can be achieved both cost-neutrally and with benefit to the environment. Here, we found that improving accordance to 4 of the 8 DASH food groups—by reducing intake of red and processed meats and dietary sodium and increasing intake of whole grains, nuts, and legumes—achieved both ends.

Although modeling studies have demonstrated the feasibility of diets that meet cost, nutrition, and environmental criteria in the United Kingdom (25), a recent observational study identified existing diet patterns that met these criteria in the French population (41). Those diets achieved lower GHGs and cost in part by having lower total energy intake, because energy intake is strongly and positively associated with dietary GHGs [see Vieux et al. (30) and Figure 1]. Furthermore, they also emphasized starchy plant-based foods and were lower in energy density, characteristics similar to DASH (42, 43). Notably, diets meeting these criteria were 17–19% lower in GHGs relative to the average French diet (41), a difference similar to the one we report between the top and bottom quintiles of DASH accordance. Altogether, this body of literature indicates that shifting population diets to lower their GHG impact may be achieved by both reducing excess energy intake and changing diet composition to strike a balance between health, environmental impact, and consumer costs.

### Methodologic considerations and limitations

This descriptive study faced a number of limitations, including being based on a cohort of older adults who had limited socioeconomic and demographic heterogeneity. Further limitations stem from a reliance on FFQs, which have a number of biases (42). There was limited detail in the available food-level GHG data, and confidence intervals in our estimates are likely to be underestimated because uncertainty around the GHG emissions of individual foods was not incorporated into the results. Similar considerations apply to our methods for deriving dietary costs, which were obtained by using retail food price data, an approach described above, that, on average, produces estimates that are lower than actual food expenditures (20). Finally, dietary costs as defined here were only a reflection of the retail price of the foods consumed and did not reflect the potential economic externalities of diet, including (for example) the health care costs to society arising from the consumption of low-price but less healthy diets. Despite these limitations, we believe this study represents an important step forward by linking food-level variables with a large, population-based sample to quantitatively examine the environmental and economic implications of the DASH diet in the United Kingdom.

### Conclusions

In conclusion, population-based strategies to preventing obesity and chronic disease need to incorporate the promotion of evidence-based dietary guidance (44). The DASH diet is one evidence-based dietary pattern that, if widely adopted, could have substantial beneficial impacts for population health as well as having beneficial effects on diet-related GHG production. Although some of the DASH food groups appear to be cost-neutral or even cost-saving, a remaining challenge will be to make all components of the DASH diet pattern more affordable to consumers.

## Supplementary Material

Supplemental data

## Footnotes

6 Abbreviations used: CO_2_eq, carbon dioxide equivalents; DASH, Dietary Approaches to Stop Hypertension; EPIC, European Prospective Investigation into Cancer and Nutrition; EPIC-Norfolk, European Prospective Investigation into Cancer and Nutrition cohort study in Norfolk; FFQ, food-frequency questionnaire; GHG, greenhouse gas; Q1, quintile 1; Q5, quintile 5.
