# Supplementary material for: Greater accordance with the Dietary Approaches to Stop Hypertension dietary pattern is associated with lower diet-related greenhouse gas production but higher dietary costs in the United Kingdom
Source: Am J Clin Nutr. 2015 Apr 29;102(1):138–45. doi: 10.3945/ajcn.114.090639 (PMC4480663; doi:10.3945/ajcn.114.090639)
Supplement: Supplemental data [file supp_102_1_138__index.html]

Supplemental data 

# Greater accordance with the Dietary Approaches to Stop Hypertension dietary pattern is associated with lower diet-related greenhouse gas production but higher dietary costs in the United Kingdom

## Supplemental data

- Supplemental data - Methods and Tables 1 and 2
