# Supplementary material for: Greater accordance with the Dietary Approaches to Stop Hypertension dietary pattern is associated with lower diet-related greenhouse gas production but higher dietary costs in the United Kingdom
Source: Am J Clin Nutr. 2015 Apr 29;102(1):138–45. doi: 10.3945/ajcn.114.090639 (PMC4480663; doi:10.3945/ajcn.114.090639)
Supplement: Supplemental data [file 114.090639_ajcn090639SupplementaryData1.docx]

*Online Supplementary Materials*

**Greater accordance with the DASH dietary pattern is associated with lower diet-related greenhouse gas production but higher dietary costs in the United Kingdom**

Pablo Monsivais, Peter Scarborough, Tina Lloyd, Anja Mizdrak, Robert Luben, Angela A Mulligan, Nicholas J Wareham, James Woodcock.

Includes

Supplementary **Methods**

Supplementary Table **S1**

Supplementary Table **S2**

**Supplementary Methods**

**Subjects**

EPIC-Norfolk participants were part of a prospective population study of 25,639 men and women aged 39–79 years, 99.5 per cent white (as self-defined on questionnaire), residing in Norfolk, UK (1). Norfolk is a county in the UK encompassing a wide socioeconomic and urban-rural distribution. The Norfolk cohort was comparable to national UK population samples with respect to characteristics including anthropometry, blood pressure, and lipids, but with a lower prevalence of current smokers than the general population of England.

**DASH accordance score**

The food group-based score is similar to one Fung and colleagues previously applied to the Nurses’ Health Study food frequency questionnaire (FFQ) (2). That score was based on consumption of 8 food groups and nutrients: fruits, vegetables (excluding potatoes), nuts and legumes, whole grains, low-fat dairy, red and processed meats, sweetened beverages and sodium. There were two key differences between that score and the DASH accordance score we applied to the EPIC cohort.

First, Fung and colleagues included peas in the *nuts and legumes* category but to be consistent with previous classifications of the EPIC FFQ data, the FFQ lines relating to peas and green beans were included in the *vegetables* category as the foods underlying these items in the EPIC FFQ were fresh, canned, frozen peas and green beans. In contrast, we assigned dried beans and dried peas to the nuts and legume category.

Another difference was in the *sweetened beverages* food group (2). The closest match in the EPIC FFQ was “fizzy soft drinks e.g. cola, lemonade”. Other beverage categories in the EPIC FFQ could not be included because they did not discriminate between those with added sugars and those with artificial sweeteners (i.e., ‘diet’ beverages). Furthermore, 64 percent of the sample were non-consumers of fizzy soft drinks. As a result, the *sweetened beverages* food group was broadened to encompass more sources of added sugars in the British diet. These other sources included non-alcoholic beverages, cereals and cereal products (mainly biscuits, buns, cakes, pastries and fruit pies) and sugar, preserves and confectionery (3). Thus, a food group consisting of the FFQ data on confectionary, sweetened cereal products such as sweet biscuits, sweet pastries, buns, fizzy drinks (non-diet) plus sugar added to hot drinks and breakfast cereals was created. This category, referred to as ‘foods high in added sugars’ had less than 1 per cent of non-consumers in the sample.

For each of the eight DASH food groups, quintiles were derived that were adjusted for energy based on the residual method (4).

**Calculation of dietary GHG emissions**

For each of the 289 food codes represented in the FFQ’s food and nutrient database, we estimated the GHG emissions as the quantity of three GHGs weighted by their global warming potential over a 100 year period. The GHG parameters were sourced from Audsley et al. (5), which estimated comparable GHG emissions for 94 food commodities consumed in the UK. The GHGs were carbon dioxide, methane and nitrous oxide. The GHG emissions for the 289 food codes were then constructed from these 94 parameters using representative ingredients lists and adjustments for density. The UK food composition tables (6), which provides nutritional data for the FFQ, were used as the first source for identifying ingredients in mixed foods and dishes. If no recipe was found then a Google search was performed with the food code name as the search term and the top website found with a recipe for the food was used. Where ingredients were identified that were not comparable to a food commodity (e.g. flaky pastry), then a recipe was identified for the ingredient using the same method. For each food code, the weights of the ingredients were added in the order of heaviest to lightest, and a 90% threshold was used such that once a value greater than 90% was achieved the remaining ingredients were disregarded. This threshold was applied to remove small ingredients with unknown GHG emissions from the calculations (e.g. ‘a pinch of nutmeg’).

For cheese, fruit juices, dried fruit and soya milk, the weight of food that is consumed is not equivalent to the weight of food that is produced. GHG emissions parameters for these food categories were not available, so we applied adjustment factors to their primary commodity to account for the change in weight between production and consumption. Where possible these adjustment factors were taken from published life cycle analyses, but when these were unavailable we used information from specific food products. For the remaining food codes a recipe was sought which was used to split the weight of the food between food commodities. This split was then used to construct a GHG emissions estimate using the GHG parameters of the original commodities.

Different GHG parameters are estimated for foods produced in the UK, the EU and outside the EU. Using 2007 food balance sheet data from the FAO (7), we produced single UK estimates of GHG emissions for each food commodity, weighted by import and export patterns. For seven food codes (drinking chocolate, chocolate digestive biscuits, semi-sweet biscuits, Coffeemate™, Horlicks™ powder, mixed toffees, and soft drinks) the use of a recipe seemed implausible given that these products are largely produced on an industrial scale. Here recipes were estimated (using percentages in the ingredients list where available). A complete spreadsheet showing recipes and calculations for all 289 food codes is available upon request.

**Supplementary Table S1**

Descriptive statistics of demographic and socioeconomic characteristics for British adults in the EPIC-Norfolk cohort

|  | **Men**  **n=10,980** | **Women**  **n=13,313** | **Overall**  **n=24,293** |
| --- | --- | --- | --- |
| **Age (yrs) @ 1HC or consent** | 59.7 | 58.9 | 59.2 |
|  |  |  |  |
| **Occupational Social Class, no. (%)^1^** |  |  |  |
| Unskilled | 307 (3) | 499 (4) | 806 (3) |
| Partly-skilled | 1416 (13) | 1727 (13) | 3143 (13) |
| Skilled occupations – manual | 2722 (25) | 2756 (21) | 5478 (23) |
| Skilled occupations – non manual | 1361 (13) | 2591 (20) | 3952 (17) |
| Managerial and Technical | 4171 (39) | 4576 (35) | 8747 (37) |
| Professional | 822 (8) | 845 (7) | 1667 (7) |
|  |  |  |  |
| **Educational Attainment, no. (%)^2^**  *years in education* |  |  |  |
| < 11 years | 3314 (30) | 5557 (42) | 8871 (37) |
| 11 – 13 years | 942 (9) | 1545 (12) | 2487 (10) |
| >13 but <16 years | 5034 (46) | 4764 (36) | 9798 (40) |
| ≥16 years | 1681 (15) | 1441 (11) | 3122 (13) |
|  |  |  |  |
|  |  |  |  |
| **Smoking status, no. (%)^3^** |  |  |  |
| Current smoker | 1295 (12) | 1480 (11) | 2775 (12) |
| Former smoker | 5936 (54) | 4236 (32) | 10172 (42) |
| Never smoker | 3674 (34) | 7473 (57) | 11147 (46) |
|  |  |  |  |
| **Total energy (Kcal/d)** | 2191 | 1928 | 2047 |
|  |  |  |  |
| ^1^ Based on Registrar General’s classification, 500 missing cases (2.1%); ^2^15 missing cases (0.1%); ^3^ 199 missing cases (0.8%). | | | |

**Supplementary Table S2**

Diet cost for quintiles of DASH diet accordance, stratified by sex. Analyses are adjusted for age and dietary energy. Data from the EPIC-Norfolk cohort.

|  | **Diet Cost, mean £/d (95% CI)** | | | | |
| --- | --- | --- | --- | --- | --- |
|  | **n** | **Men** |  | **n** | **Women** |
| **Q1 (lowest accordance)** | 2028 | 3.67 (3.64 to 3.70) |  | 2865 | 3.81 (3.77 to 3.84) |
| **Q2** | 2216 | 3.87 (3.84 to 3.90) |  | 2637 | 4.02 (3.98 to 4.05) |
| **Q3** | 2457 | 3.99 (3.94 to 4.03) |  | 2914 | 4.16 (4.13 to 4.20) |
| **Q4** | 2041 | 4.10 (4.06 to 4.13) |  | 2447 | 4.31 (4.28 to 4.34) |
| **Q5 (highest accordance)** | 2238 | 4.28 (4.23 to 4.33) |  | 2450 | 4.52 (4.49 to 4.56) |
| Difference Q5/Q1 (%) |  | +16.6 |  |  | +18.6 |
| P-value, Q1 versus Q5^1^ |  | <0.001 |  |  | <0.001 |
| P-value, trend^2^ |  | <0.001 |  |  | <0.001 |
| ^1^Based on post-hoc, pairwise test comparing highest and lowest levels; ^2^Based on linear regression with quintiles of DASH accordance treated as a group linear variable. | | | | | |

**References**

1. Day N, Oakes S, Luben R, Khaw KT, Bingham S, Welch A, Wareham N. EPIC-Norfolk: study design and characteristics of the cohort. European Prospective Investigation of Cancer. Br J Cancer 1999;80 Suppl 1:95-103.

2. Fung TT, Chiuve SE, McCullough ML, Rexrode KM, Logroscino G, Hu FB. Adherence to a DASH-style diet and risk of coronary heart disease and stroke in women. Arch Intern Med 2008;168(7):713-20. doi: 168/7/713 [pii] 10.1001/archinte.168.7.713.

3. Bates B, Lennox A, Swan G. National diet and nutrition survey. Headline results from year 1 of the rolling programme (2008/2009). FSA and the DH: London 2010.

4. Willett W. Nutritional epidemiology. 3rd ed. ed. New York ; Oxford: Oxford University Press, 2013.

5. Audsley E, Brander M, Chatterton J, Murphy-Bokern D, Webster C, Williams A. How low can we go? An assessment of greenhouse gas emissions from the UK food system and the scope for reduction by 2050. WWFFCRN. http://www. fcrn. org. uk, 2009.

6. McCance RA, Holland B, Widdowson EM, Royal Society of Chemistry (Great Britain), Great Britain. Ministry of Agriculture Fisheries and Food. McCance and Widdowson's the composition of foods. 5th rev. and extended ed. Cambridge, UK: Royal Society of Chemistry : Ministry of Agriculture, Fisheries and Food, 1993.

7. Food and Agriculture Organization of the United Nations; Food Balance Sheets (Web page): http://faostat3.fao.org/browse/FB/FBS/E.
